# Contrasting processing tomato cultivars unlink yield and pollen viability under heat stress

**DOI:** 10.1093/aobpla/plab046

**Published:** 2021-07-17

**Authors:** Golan Miller, Avital Beery, Prashant Kumar Singh, Fengde Wang, Rotem Zelingher, Etel Motenko, Michal Lieberman-Lazarovich

**Affiliations:** 1 Institute of Plant Sciences, Agricultural Research Organization – Volcani Center, Rishon LeZion 7505101, Israel; 2 Department of Biotechnology, Mizoram University (A Central University), Pachhunga University College Campus, Aizawl 796005, Mizoram, India; 3 Institute of Vegetables and Flowers, Shandong Academy of Agricultural Sciences, Jinan 250100, China; 4 Université Paris-Saclay, INRAE, AgroParisTech, Economie Publique, 78850 Thiverval-Grignon, France

**Keywords:** Field conditions, pollen quality, productivity, stress response, thermotolerance

## Abstract

Climate change is causing temperature increment in crop production areas worldwide, generating conditions of heat stress that negatively affect crop productivity. Tomato (*Solanum lycopersicum*), a major vegetable crop, is highly susceptible to conditions of heat stress. When tomato plants are exposed to ambient day/night temperatures that exceed 32 °C/20 °C, respectively, during the reproductive phase, fruit set and fruit weight are reduced, leading to a significant decrease in yield. Processing tomato cultivars are cultivated in open fields, where environmental conditions are not controlled; therefore, plants are exposed to multiple abiotic stresses, including heat stress. Nonetheless, information on stress response in processing tomatoes is very limited. Understanding the physiological response of modern processing tomato cultivars to heat stress may facilitate the development of thermotolerant cultivars. Here, we compared two tomato processing cultivars, H4107 and H9780, that we found to be constantly differing in yield performance. Using field and temperature-controlled greenhouse experiments, we show that the observed difference in yield is attributed to the occurrence of heat stress conditions. In addition, fruit set and seed production were significantly higher in the thermotolerant cultivar H4107, compared with H9780. Despite the general acceptance of pollen viability as a measure of thermotolerance, there was no difference in the percentage of viable pollen between H4107 and H9780 under either of the conditions tested. In addition to observations of similar pollen germination and bud abscission rates, our results suggest that processing tomato cultivars may present a particular case, in which pollen performance is not determining reproductive thermotolerance. Our results also demonstrate the value of combining controlled and uncontrolled experimental settings, in order to validate and identify heat stress-related responses, thus facilitating the development of thermotolerant processing tomato cultivars.

## Teaser

Our paper deals with the response of tomato plants to high temperatures. Tomato is an important crop, and tomato plants are very sensitive to high temperatures. Therefore, the increase in temperatures due to climate change poses a threat to tomato production. We found that a specific tomato cultivar is heat stress-tolerant, with better productivity under high temperatures. Usually, such tolerance is linked with pollen grains viability. Interestingly, in our case, pollen characteristics were eliminated as a causal factor for heat stress tolerance, meaning that other factors are involved. Follow-up studies are performed towards the development of heat-tolerant tomato.

## Introduction

Plant physiology and development are prominently affected by changes in ambient temperatures. Due to the global climate change, temperatures are gradually shifting and temperature extremes occur more frequently. Predictions of the effect of temperature increment on major crops yield show that each 1 °C increase in global mean temperature would cause yield reduction by 3.1–7.4 % on average (Zhao *et al.* 2017). Recent IPCC reports estimate global warming is likely to reach a 1.5 °C increase in average surface temperature between 2030 and 2052 if it continues to increase at the current rate, and reach a 2–4 °C increase by the end of the 21st century (Masson-Delmotte *et al.* 2018), thus challenging crop productivity and food security. Yield reduction due to heat stress was documented in various crops such as cereals (wheat [*Triticum aestivum*], rice [*Oryza sativa*], barley [*Hordeum vulgare*], sorghum [*Sorghum bicolor*] and maize [*Zea mays*]), pulses (chickpea [*Cicer arietinum*]) and oil yielding crops (mustard [*Sinapis alba*], canola [*Brassica napus*]), fruits and vegetables (potato [*Solanum tuberosum*], eggplant [*Solanum melongena*], cabbage [*Brassica oleracea*], cauliflower [*Brassica oleracea*], lettuce [*Lactuca sativa*], onion [*Allium cepa*], cucumber [*Cucumis sativus*], musk melon [*Cucumis melo*], watermelon [*Citrullus lanatus*] and pumpkin [*Cucurbita pepo*]; Hasanuzzaman *et al.* 2013). When heat stress occurs during the reproductive phase of plant development, the observed consequences include morphological alterations of anthers, style elongation, bud abscission and reduced fruit number, size and seed set. The development of pollen is considered the most heat-sensitive stage (Lohani *et al.* 2020) as it was shown to be more sensitive than both the sporophyte and female gametophyte tissues (Peet *et al.* 1998; Young *et al.* 2004; Wang *et al.* 2019). Heat stress disrupts meiotic cell division, pollen morphology and size, and grain number, viability and germination capacity (Peet *et al.* 1998; Pressman *et al.* 2002; Firon *et al.* 2006; Prasad *et al.* 2006; Endo *et al.* 2009; Djanaguiraman *et al.* 2013; Giorno *et al.* 2013; Begcy *et al.* 2019), leading to male sterility and reduced fruit/grain production.

Tomato (*Solanum lycopersicum*), an important vegetable crop worldwide, cultivated in a wide range of agro-climatic regions, is very sensitive to heat stress. The tomato fruit set is optimal when the average day and night temperatures range between 21–29 °C and 18–21 °C, respectively (Pelzer 2008). Prolonged stress of day temperatures exceeding 32 °C with night temperature above 20 °C cause reduced fruit set, fruit weight, total yield and seed production (El-Ahmadi and Stevens 1979; Peet *et al.* 1998; Sato *et al.* 2000; Firon 2006). Pollen heat stress-related damage in tomato, exhibited by morphological alterations and reduced pollen viability and germination rates, was observed after short episodes of high temperatures at 40 °C, or after chronic exposure to milder heat stress of 31–32 °C/25–28 °C day/night for several months (Iwahori 1966; Firon *et al.* 2006; Giorno *et al.* 2013). The decrease in pollen viability and/or germination was shown to cause a significant decrease in fruit set; therefore, pollen viability was used as a screening approach to identify heat stress-tolerant tomato genotypes (Iwahori 1965; Rudich *et al.* 1977; Abdul-Baki 1992; Sato *et al.* 2000). For example, the thermotolerant tomato cultivar LA1994 was shown to have high pollen viability during heat stress, which correlated with high yield under controlled conditions (Zhou *et al.* 2017). Additionally, several cultivars that present high pollen viability under heat stress conditions in a controlled temperature environment set more fruit during thermal stress in the field (Poudyal *et al.* 2019). Consequently, several tomato genotypes were identified that maintain a higher level of pollen viability under heat stress conditions (Dane *et al.* 1991; Paupière *et al.* 2017; Driedonks *et al.* 2018). Pollen viability is therefore often used as a measure of thermotolerance, establishing the correlation between pollen viability and fruit set (Firon *et al.* 2006; Xu *et al.* 2017; Pham *et al.* 2020; Rutley *et al.* 2021).

In contrast to the wealth of data demonstrating the correlation between pollen heat stress damage and fruit set, examples of heat stress tolerance/sensitivity not correlated with pollen viability are very scarce. Gonzalo *et al.* (2020) performed a wild species population screen for reproductive traits under heat stress conditions, and no correlation was found between pollen viability and fruit set (Gonzalo *et al.* 2020). In a more recent study, Ayenan *et al.* (2021) screened a collection of 42 cultivated and wild tomato genotypes with good yield components under long-term mild heat stress and did not find association between the proportion of viable pollen and fruit set (Ayenan *et al.* 2021). In this paper, we present yet another example for heat stress tolerance that is not correlated with pollen viability, in a processing cultivar of tomato.

Tomato processing cultivars are used as raw material for the food industry. Thus, breeding companies developed cultivars suited for mechanical harvesting and canning processes. These cultivars are characterized by a determinate growth habit, synchronized fruit set and firm flesh (Hanna 1971; Gould 1992). Processing tomato plants are cultivated only in open fields, where heat stress conditions are prevalent. However, information regarding the response of processing cultivars to heat stress is very limited.

Here, we characterized the heat stress response of two processing tomato cultivars, which are typically grown in open field conditions therefore exposed to a combination of stress factors, including heat stress, during the reproductive stage. We show that the constant difference in yield between these cultivars is attributed to high-temperature conditions. In order to gain information specifically for the response to heat stress, the same cultivars were tested in a controlled greenhouse, under heat stress and control conditions in a parallel set-up. This set-up allows the identification of specific heat stress-related traits, which is not possible under the uncontrolled, multi-stress field conditions.

## Materials and Methods

The experimental design is graphically summarized in **Supporting Information—Fig. S1**.

### Plant material and growth conditions

Two tomato commercial processing cultivars: H4107 and H9780, were obtained from Green Seeds Ltd. These hybrid cultivars were developed by the Heinz Company (https://d36rz30b5p7lsd.cloudfront.net/372/studio/assets/v1611911409263_1054604699/2021%20HeinzSeed%20International%20Brochure.pdf). H9780 was released in 2001, as a full season paste tomato suitable for peel/dice applications, adapted for arid climates. It is characterized by large fruit, semi-prostrate vine, high °Brix and good colour. H4107 was released in 2009, a mid-season hybrid peel/dice tomato with low viscosity, medium vine with excellent cover, smooth oval fruit (Wehner and Mou 2019).

H4107 and H9780 were grown during 2018 in three different experimental fields, across different environments as follows: (i) ‘Upper Galilee’ site, at the Northern part of Israel (33°10′50.6″N latitude, 35°34′49.6″E longitude; field size 100 plants), (ii) ‘Eden’ site (32°27′58.2″N latitude, 35°29′12.2″E longitude; field size 80 plants) and (iii) ‘Volcani’ site at a central region of Israel (31°59′34.6″N latitude, 34°49′01.8″E longitude; field size 40 plants). The Upper Galilee field is located in a region that is characterized by hot days and cooler nights during the processing tomato season (May–July), whereas the ‘Eden’ field is located in the Jordan Valley which is characterized by high day and night temperatures, and high humidity. For this reason, planting in Eden starts earlier (February–May), to avoid extreme heat stress and yield losses. In addition, we set a small experimental field at the Volcani Center Agricultural Research Organization (ARO), located in a more temperate region. The two cultivars were grown in a completely randomized design in 3–5 replicas (plots) per field. Seeds were sown in germination trays and transplanted in open fields after 3 weeks. Mature plants were maintained under standard horticultural practices. During the whole growing period climatic data were recorded using the weather stations ‘Khavat Eden’, ‘Beit Dagan’ and ‘Mop Tzafon’ located in Eden, Volcani and Upper Galilee fields, respectively **[see****Supporting Information—Table S1****]**. In addition, the two cultivars were grown in climate-controlled greenhouses at the Naan site of Evogene Ltd Company **[see****Supporting Information—Table S2****]**. In the controlled experiment, four plants from each cultivar were grown under moderate chronic heat stress (MCHS) conditions (32 °C/22 °C day/night, starting at flowering) or control conditions (25 °C/18 °C day/night), in a randomized set-up, identical between the two rooms.

### Reproductive traits evaluation

Fruit set and fruit production were evaluated in all three experimental fields and in the controlled experiment performed during 2018. Fruit production (fruit weight) was evaluated by weighing total red-ripe fruits per repeat (plot or plant in the field or controlled experiments, respectively). Fruit set was evaluated from 10 randomly selected inflorescences from each plot in the field experiments. In the controlled experiment, fruit set was evaluated from three randomly selected inflorescences from four different plants (12 inflorescences per cultivar). Fruit set was calculated as follows: $\frac{Total\;no.\;\;of\;fruits\;per\;inflorescence}{Total\;no.\;\;of\;flower\;positions\;per\;inflorescence}*100=Fruit\;set\;ratio$. Seed number per fruit was examined by seeds extraction using three fruits from five plants (Volcani field) or three fruits from five plots (Upper Galilee field). In the controlled experiment, 5–25 fruits from all four plants were sampled. Seeds were extracted using sulfuric acid; the locular gel containing the seeds was extracted and soaked in 2 % sulfuric acid solution. After 3 h, the seeds were transferred into a net bag and rinsed with tap water. Seeds were then thoroughly dried in open air for a few days. Seed number per fruit was calculated by weighing a small portion that was manually counted. Then, the total amount of seeds was estimated by weighing and calculating. Bud abscission was calculated as the rate of buds drop per inflorescence, using 10 randomly selected inflorescences from each plot in the field experiments.

### Pollen viability analysis

For pollen viability analysis, conducted during field and controlled experiments during 2018, flowers at anthesis were collected in the morning (7:00–10:00 am). In total, three flowers per plant were collected and three plants were used per cultivar. Each anther was cut into two pieces and put in a 1.5-mL tube filled with 0.5 mL germination solution (1 mM KNO_3_, 3 mM Ca(NO_3_)_2_·4H_2_O, 0.8 mM MgSO_4_·7H_2_O, 1.6 mM boric acid [H_3_BO_3_]; Pressman *et al.* 2002) followed by adding 20 μL of Alexander dye (20 mL of ethanol, 20 mg of malachite green, 50 mL of distilled water, 40 mL of glycerol, 100 mg of acid fuchsin, 2 g of phenol and 2 mL of lactic acid for a 100 mL solution; Alexander 1980). Samples were observed under a Leica DMLB epi-fluorescence microscope (Germany) using a Bright Field filter, magnified by 10–20. Three fields containing representative pollen patterns were captured with a DS-Fi1 digital camera using NIS-Elements BR3.0 software (Nikon). Viable (purple) and non-viable (blue–green) pollen grains were counted manually with ImageJ version 1.43 software using the ‘Cell counter’ plugin (Schneider *et al.* 2012).

### Pollen germination analysis

In order to test pollen germination, a pool of three open flowers (from three different plants) was collected from each plot in the field experiments. Flowers were dried for 1 h. Dried anthers were transferred to 0.5 mL of liquid germination media (20 mg H_3_BO_3_, 60 mg CaNO_3_, 40 mg MgSO_4_, 20 mg KNO_3_, 10 g sucrose, 100 mL double distilled water) and were vortexed vigorously for 10 s. SeaKem® LE Agarose (LONZA Company) was added to the liquid media, dissolved and poured onto a microscope slide, flatted with a Parafilm® tape and another slide on top. After solidification, slides were transferred to a dark humid chamber. Then, pollen solution was transferred to the solid media and incubated for 1.5 h. Slides were analysed using a DM500 Leica microscope. Pollen germination was pictured and counted using the ImageJ software (Fiji).

### Statistical analysis

One-way ANOVA was employed to identify significant differences (*P* < 0.05) between the cultivars for each trait. When ANOVA identified significant differences among genotypes, we used the Student’s *t*-test method for all differences between means. These conservative procedures limited the probability of rejecting a true null hypothesis to the desired (*P* < 0.05) level. All statistical analyses were performed using JMP Version 3.2.2 (SAS Institute, Inc., Cary, NC, USA).

## Results

### Consistent difference in yield between H4107 and H9780 across multiple years and locations

**Figure 1.**
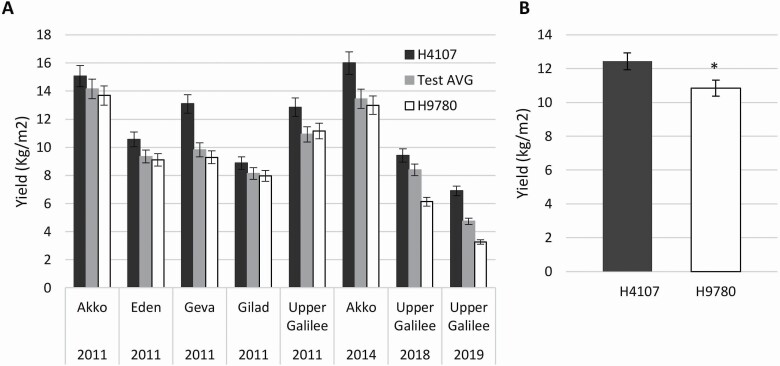
Consistent difference in yield between H4107 and H9780 across years and locations. (A) Average yield of H4107 and H9780 in years and locations testing both cultivars. The test average obtained by yield measurements of multiple cultivars is presented as well. (B) Average yield of H4107 and H9780 across years and locations presented in (A). *Statistically significant difference (*P*-value < 0.05).

Following a survey of processing tomato field-testing data from 15 years (2005–19) across 17 different locations, we detected a consistent difference between two cultivars, i.e. H4107 and H9780 **[see****Supporting Information—Table S3****]**. While the yield of H4107 was always above the test average, the yield of H9780 was always lower than the test average **[see****Supporting Information—Table S3****]**. When we compared the results of specific years and locations where both cultivars were tested simultaneously, the average yield was 12.4 and 10.8 k/m^2^ for H4107 and H9780, respectively, providing a significant difference (Fig. 1A and B).

### The difference in yield between H4107 and H9780 is associated with high-temperature conditions

**Figure 2.**
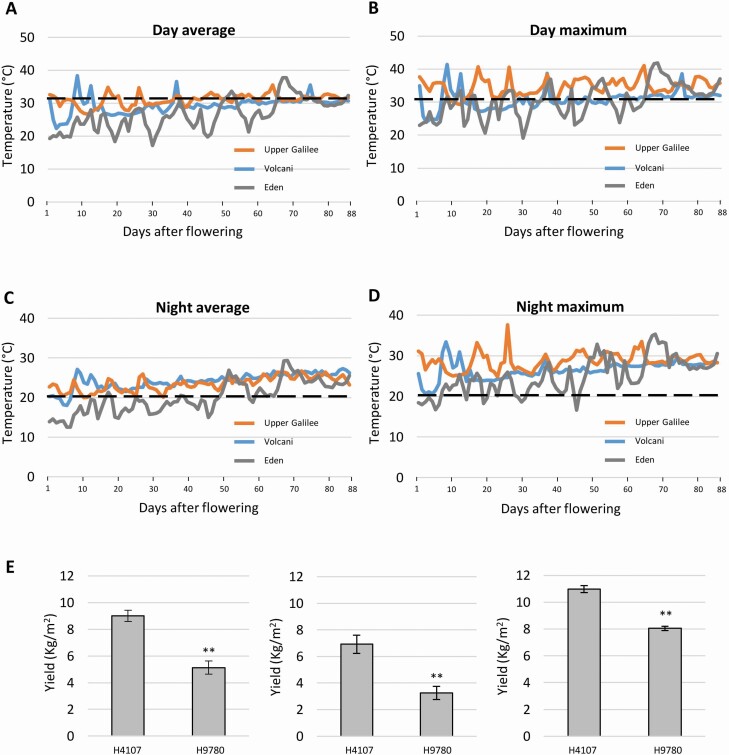
Field experiment conditions and yield. Temperatures were recorded constantly in the three experimental sites: Upper Galilee, Volcani and Eden. Daily average (A), daily maximum (B), night average (C) and night maximum (D) were calculated for the reproductive period and are presented from the first day of flowering until the end of the experiment (88 days after flowering). (E) Yield performance for H4107 and H9780 in the Volcani (left), Upper Galilee (middle) and Eden (right) fields. **Statistically significant difference (*P*-value < 0.01).

**Figure 3.**
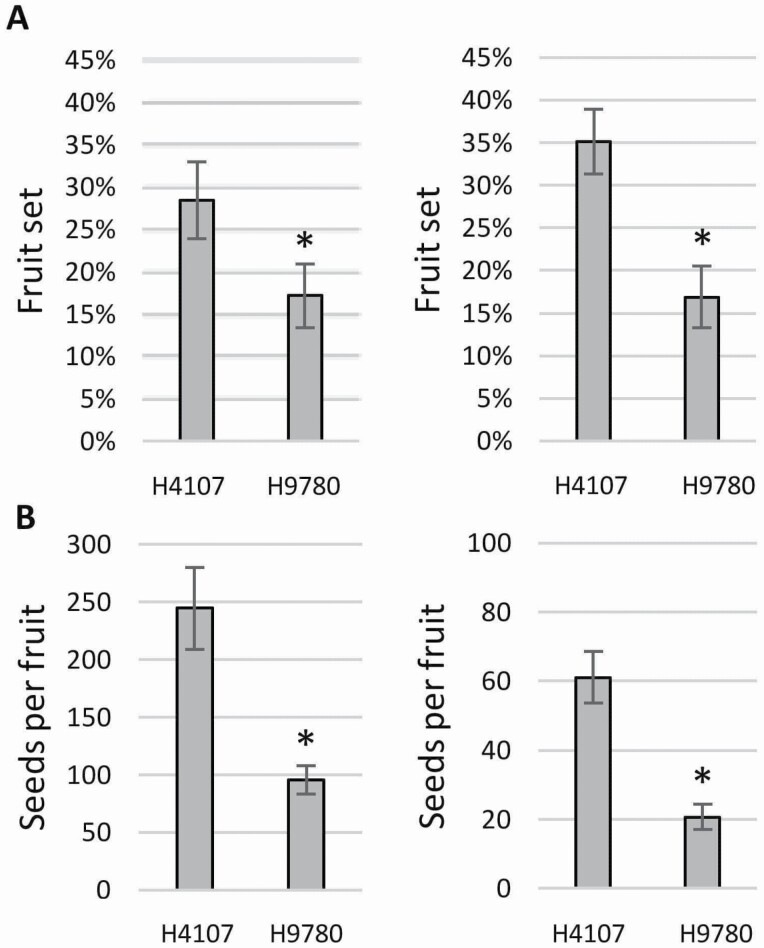
Fruit set and seed number measurements in the field experiments during 2018. (A) Fruit set of H4107 and H9780 in Upper Galilee (left) and Volcani (right) fields. (B) Seed number per fruit for H4107 and H9780 in Upper Galilee (left) and Volcani (right) fields. *Statistically significant difference (*P*-value < 0.05).

To test whether the observed difference in yield between H4107 and H9780 is due to their differential response to high temperatures, we set field experiments in two locations that are routinely used for processing tomato cultivation, however, differing by their environmental conditions. In addition, we set a small experimental field at the Volcani Center, in close proximity to the lab. Overall, we tested the plants under field conditions in three different environments. Environmental data were obtained for each field from a local meteorological station, enabling recording temperature every 3 h; hence, we calculated day and night average and maximum temperatures. Considering that tomato plants experience heat stress when day temperature exceeds 32 °C and night temperature exceeds 20 °C, our analysis shows that heat stress conditions were indeed prevalent in all three locations, though with some differences (Fig. 2A–D). In the Eden field, due to the early planting, heat stress conditions developed around 50 days after flowering. Nonetheless, day and night maximal temperatures surpassed threshold values already 5 days after flowering, generating heat stress conditions throughout the entire reproductive period. In the Upper Galilee field, daily average temperatures were around 32 °C, reaching a maximum of ~35 °C in most days, including three incidences of above 40 °C. Night temperatures in the Upper Galilee field were higher than 20 °C throughout the period, reaching a maximum of over 30 °C on several occasions, presenting more severe heat stress than in the Eden field. Lower temperatures were observed in the Volcani field, where the daily average was usually under 32 °C, with four exceptional heat waves. Night temperatures were still high averaging around 25 °C throughout the tested period; thus, the plants in the Volcani field also experienced heat stress conditions (Fig. 2A–D). Under the above-described conditions, we found that the yield of H4107 was significantly higher than that of H9780 in all fields (Fig. 2E), in agreement with our analysis of multiple years and locations data (Fig. 1). While H4107 produced 9.0, 6.9 and 11.0 kg fruit per m^2^ in Upper Galilee, Volcani and Eden, respectively, H9780 produced 5.1, 3.3 and 8.0 kg fruit per m^2^ in the same respective fields. Moreover, yield levels in both cultivars were higher in Eden than in the Upper Galilee and Volcani fields that experienced a more substantial heat stress, suggesting that yield levels are indeed affected by the high temperatures in these locations. The reproductive difference between H4107 and H9780 was further demonstrated by testing fruit set and seed production in the Upper Galilee and Volcani fields (Fig. 3). In these locations, H4107 reached 28 % and 35 % fruit set, respectively, while H9780 had 17 % fruit set in both locations (Fig. 3A). Similarly, H4107 produced a higher number of seeds per fruit versus H9780, reaching 244 and 96, respectively, in the Upper Galilee field. In the Volcani field, H4107 had on average 61 seeds per fruit, and H9780 produced only 21 seeds per fruit on average, maintaining a significant difference (Fig. 3B).

**Figure 4.**
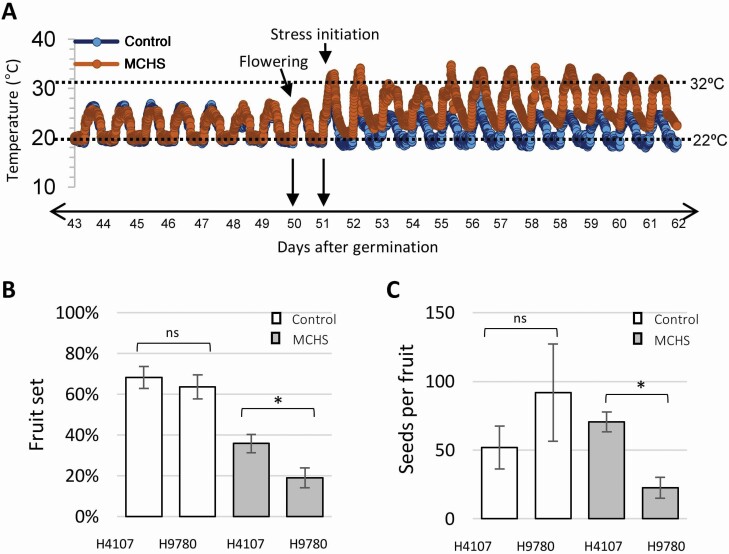
Controlled experiment conditions and reproductive measurements. (A) Temperatures measured every 5 min in both control (blue) and MCHS (brown) greenhouses. Black arrows denote day of flowering and day of stress initiation. Threshold temperatures for heat stress conditions in tomato are marked by dotted lines. (B) Fruit set for H4107 and H9780 under control (white bars) and MCHS (grey bars) conditions. (C) Seed number per fruit in H4107 and H9780 under control (white bars) and MCHS (grey bars) conditions. *Statistically significant difference (*P*-value < 0.05). ns, not significant.

In order to validate the effect of heat stress on the productivity of H4107 and H9780, we set a controlled greenhouse experiment. At the beginning of the experiment, both rooms were maintained under control conditions (25 °C/18 °C day/night). Once plants started to flower, MCHS (32 °C/22 °C day/night) was initiated in one room while the other room was kept at control conditions throughout the rest of plants growth (Fig. 4A). Fruit set and seed production were analysed under both conditions. We found no significant difference between H4107 and H9780 in both parameters measured (i.e. 64–68 % fruit set and 52–92 seeds per fruit) under control conditions. However, under MCHS conditions, H4107 performed better than H9780, as the fruit set was 36 % versus 19 % in H9780. Seed number per fruit was 71 and 23 for H4107 and H9780, respectively (Fig. 4B and C).

### The difference in heat tolerance between H4107 and H9780 is not related to pollen viability

**Figure 5.**
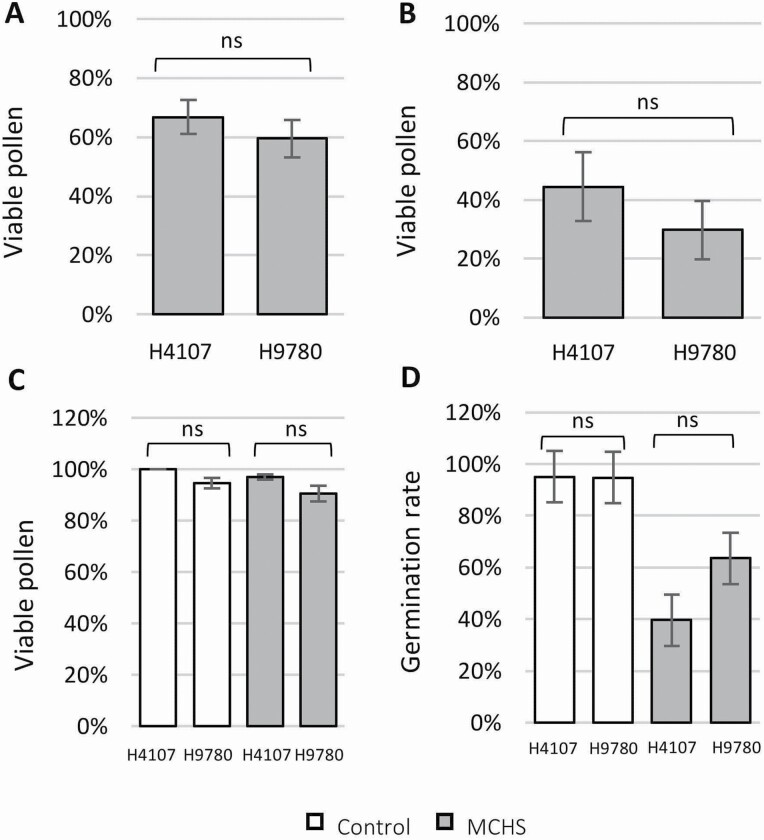
Pollen viability and germination under heat stress conditions. Percentage of viable pollen from post-anthesis flowers of H4107 and H9780 at the (A) Upper Galilee field, (B) Volcani field and (C) controlled greenhouses, under control (white bars) and MCHS (grey bars) conditions. (D) Pollen germination rate in the controlled experiment. ns, not significant.

To test whether the heat stress tolerance of H4107 can be explained by pollen viability, we analysed pollen viability percentage in field and controlled conditions. In the Upper Galilee field, we found no significant difference between H4107 and H9780, as both showed 60–70 % viable pollen out of total pollen grains (Fig. 5A). Pollen viability was lower in the Volcani field (30–45 %), yet still similar between the cultivars (Fig. 5B). In the controlled experiment, pollen viability reached as high as 90–100 %, even under MCHS conditions, and again, similar between H4107 and H9780. Interestingly, the same levels were found under control conditions (Fig. 5C). When the rate of pollen germination was evaluated in the controlled experiment we found that although the stress effect is evident, still no difference was detected between H4107 and H9780 (Fig. 5D).

## Discussion

Current literature on processing tomatoes in general and on their response to heat stress in particular is very limited. Here, we identified a consistent difference in yield between two processing cultivars: H4107 and H9780, across multiple years and locations. This difference is manifested by higher fruit set and total fruit weight of H4107. Both H4107 and H9780 were bred and adapted for humid and arid environments by the Heinz Company, but heat stress tolerance was not addressed so far. We aim to understand the source of this difference in order to promote breeding for high yield in field-grown processing tomatoes. Since the field environment imposes various stresses to the plants, and tomato being particularly sensitive to elevated temperatures, we set to test the possibility that high-temperature conditions are causing the observed difference in yield. We found that H4107 is more heat stress-tolerant than H9780, presenting better reproductive performance in terms of fruit set and seed production under high-temperature conditions. Although relative humidity is known to affect fruit set and yield in tomato (Harel *et al.* 2014), this factor did not account for the difference between H4107 and H9780 as relative humidity levels were similar between control and MCHS conditions **[see****Supporting Information—Fig. S2****]**.

One of the earliest studies on heat stress response in tomato showed that bud abscission and style exertion were more pronounced in heat-susceptible cultivars, leading to low fruit set under heat stress (Levy *et al.* 1978). Later observations demonstrated that style exertion in different tomato genotypes ranges from 25 to 55 % under high-temperature conditions (Saeed *et al.* 2007). More recently, bud abscission and style exertion were correlated with reduced fruit set under field conditions as well (Kugblenu *et al.* 2013; Singh *et al.* 2015). In processing tomato cultivars, however, the phenomena of style exertion is very rare, and we did not detect it in our experiments (by visual inspection). Bud abscission was monitored in the Upper Galilee and Volcani fields, but no difference was found between H4107 and H9780 **[see****Supporting Information—Fig. S3****]**.

Pollen viability is widely recognized as a main parameter determining plant heat stress tolerance (Dane *et al.* 1991; Paupière *et al.* 2017; Driedonks *et al.* 2018). Therefore, we tested whether the heat stress tolerance of H4107 can be at least partially explained by a higher degree of pollen viability under heat stress conditions. However, our results show that there is no difference in pollen viability between H4107 and H9780 either under stressful field conditions, or under chronic heat stress imposed artificially (Fig. 5). Therefore, we conclude that the thermotolerance of H4107 is not caused by better pollen viability, nor pollen germination capabilities. We found only one publication reporting a similar observation (i.e. pollen viability not affecting fruit set and yield under heat stress) for several greenhouse tomato cultivars (Ayenan *et al*. 2021). Thus, our results suggest that while pollen viability is a valid trait demonstrating heat stress tolerance in various tomato genotypes, it may not apply to all cultivars, and special attention should be paid for processing tomato. Other factors may mediate the tolerance in this system, possibly related to female reproduction development and function and post-pollination interactions (Peet *et al.* 1997; Xu *et al.* 2017). These issues were not addressed in this study and will be a relevant direction in future studies.

Generally, in plant science research, field and greenhouse data are inconsistent, explained by the big difference in environmental conditions between the two experimental systems. In our case, fruit set was very similar between field (28–36 % and 17 % for H4107 and H9780, respectively) and controlled heat stress (36 % and 19 % for H4107 and H9780, respectively), supporting the occurrence of heat stress conditions in the field experiments **[see****Supporting Information—Table S4****]**. Importantly, these results confirm that the observed difference in yield and other reproductive traits under open field conditions are due to high temperatures. Thus, our results demonstrate consistency in regard to a complex trait (yield), suggesting that in our system, controlled greenhouse experiments are highly relevant for agricultural conditions, facilitating translating research from lab to practice. This approach is being recognized recently, with the emerging of publications testing the response to heat stress in tomato, importantly comparing greenhouse with field conditions (Poudyal *et al.* 2019; Bhattarai *et al.* 2021; Ro *et al.* 2021).

In order to address the challenge of maintaining crop productivity in areas of temperature increment, the development of thermotolerant cultivars is needed. To achieve that, a comprehensive understanding of the agronomical, physiological and molecular responses of crop plants to heat stress is vital (Berry and Bjorkman 1980; Brestic *et al.* 2018). In light of the research presented here, which demonstrates an unusual feature of specific cultivars, emphasis should be put on relevant cultivars that may offer different attributes in terms of response to the environment. Additionally, our results demonstrate the importance of temperature-controlled experimental systems in isolating specific heat stress-related phenomena.

## Supporting Information

The following additional information is available in the online version of this article—

Figure S1. Experimental design scheme. The experimental flow is described, indicating main results. In yellow: parameters in which H4107 was higher than H9780. In orange: parameters in which no significant difference was found between H4107 and H9780. FS, fruit set. SN, seed number per fruit. PV, pollen viability. Y, yield.

Figure S2. Relative humidity rate (%RH). (A) In the three experimental field sites: Volcani, Upper Galilee and Eden. Data were recorded throughout the growth period, presented are 88 days from sowing to harvest. (B) In control and moderate chronic heat stress (MCHS) greenhouses. Data were recorded throughout the growth period, a representative period of 10 days is presented.

Figure S3. Bud abscission in field experiments: (A) Upper Galilee and (B) Volcani.

Table S1. Temperature (°C) (A) and relative humidity (%RH) (B) measured in the three experimental fields in 2018, during the whole plant growth period (sowing to harvest).

Table S2. Temperature (°C) and relative humidity (%RH) measured in the controlled experiment, in the control and moderate chronic heat stress (MCHS) greenhouses.

Table S3. Yield measurements of H4107 and H9780 processing tomato cultivars from field trials that were conducted between 2005 and 2019 across different locations. The test average is also presented. na, not applicable (not tested).

Table S4. Correlation estimates between all parameters tested. Y, yield. PV, pollen viability. FS, fruit set. SN, seed number per fruit. na, not applicable. ns, non-significant.

plab046_suppl_Supplementary_Figures

plab046_suppl_Supplementary_Table_S1

plab046_suppl_Supplementary_Table_S2

plab046_suppl_Supplementary_Table_S3

plab046_suppl_Supplementary_Table_S4

## Data Availability

No data set was generated in this study.

## Notes

**Form & Function. Chief Editor**: Kate McCulloh
