# Supplementary figures and images for: Contrasting processing tomato cultivars unlink yield and pollen viability under heat stress
Source: AoB Plants. 2021 Jul 17;13(4):plab046. doi: 10.1093/aobpla/plab046 (PMC8356174; doi:10.1093/aobpla/plab046)

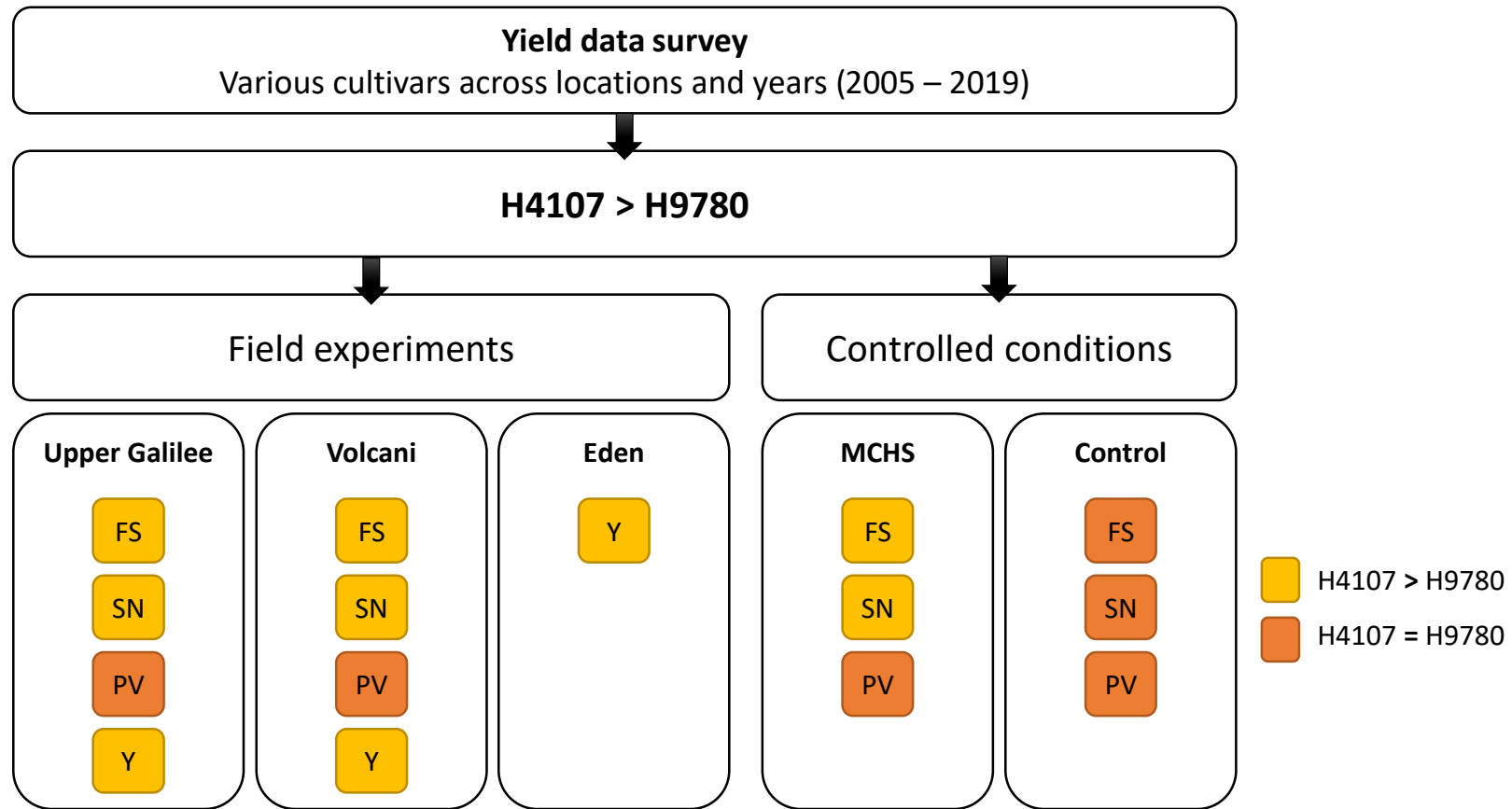

Figure S1

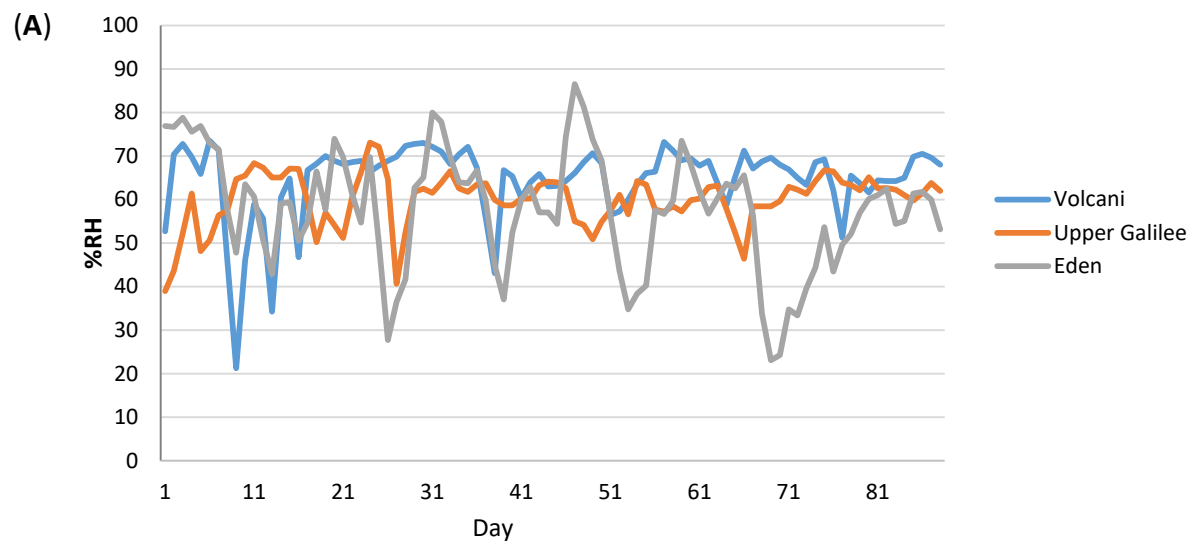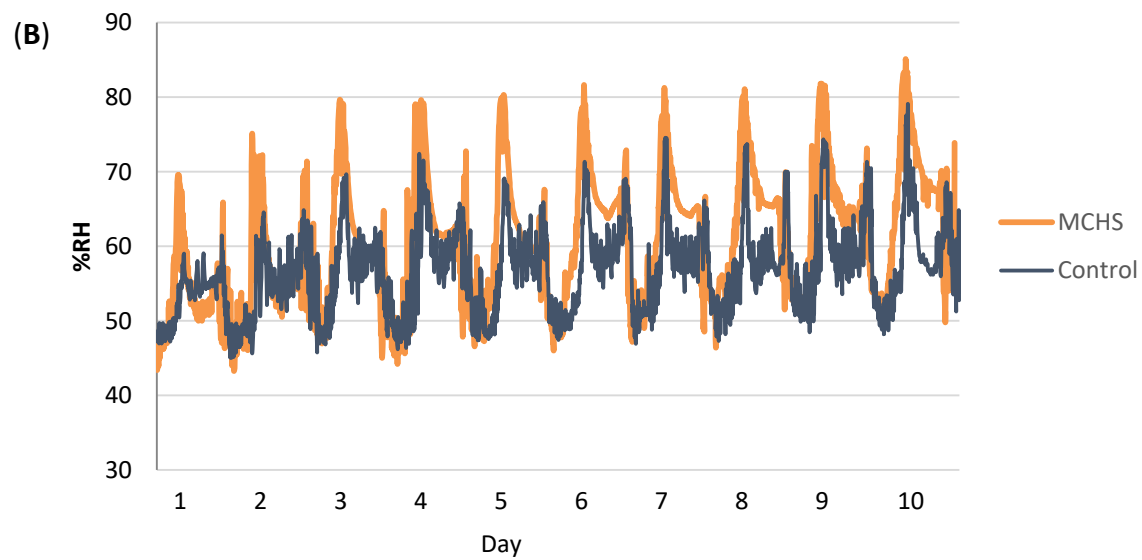

Figure S2

**(A)**

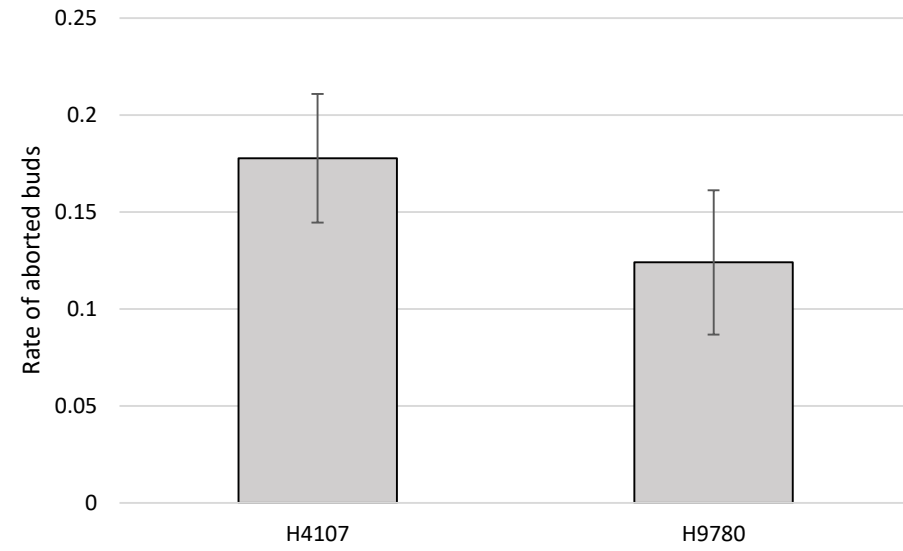

**(B)**

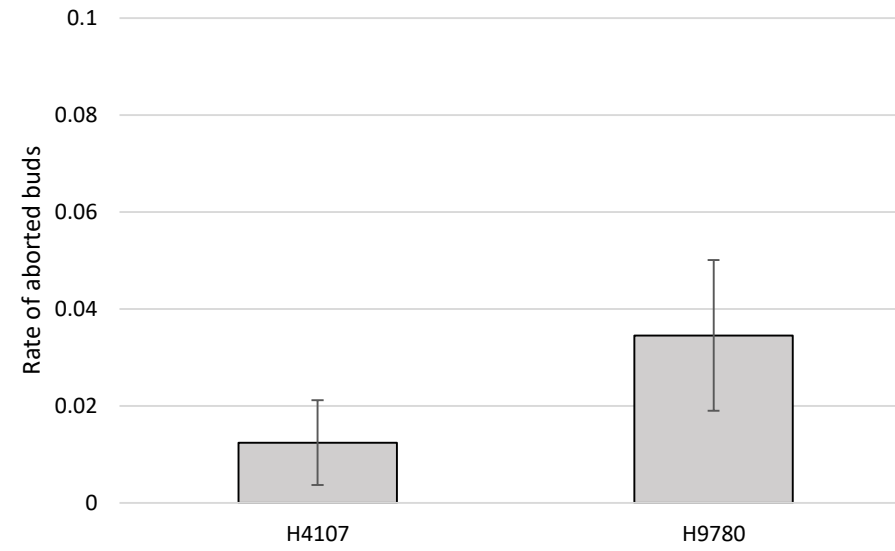

Figure S3

Supplement: plab046_suppl_Supplementary_Figures [file plab046_suppl_supplementary_figures.pdf]
